# Einstein-Podolsky-Rosen Steering for Mixed Entangled Coherent States

**DOI:** 10.3390/e23111442

**Published:** 2021-10-31

**Authors:** Sayed Abdel-Khalek, Kamal Berrada, Mariam Algarni, Hichem Eleuch

**Affiliations:** 1 Department of Mathematics and Statistics, College of Science, Taif University, P.O. Box 11099, Taif 21944, Saudi Arabia; sabotalb@tu.edu.sa; 2 Department of Physics, College of Science, Imam Mohammad Ibn Saud Islamic University (IMSIU), Riyadh 11432, Saudi Arabia; 3 The Abdus Salam International Centre for Theoretical Physics, Strada Costiera 11, 34151 Trieste, Italy; 4 Mathematical Sciences Department, College of Science, Princess Nourah bint Abdulrahman University, Riyadh 11564, Saudi Arabia; mmalgarni@pnu.edu.sa; 5 Department of Applied Physics and Astronomy, University of Sharjah, Sharjah 27272, United Arab Emirates; heleuch@sharjah.ac.ae; 6 Department of Applied Sciences and Mathematics, College of Arts and Sciences, Abu Dhabi University, Abu Dhabi 59911, United Arab Emirates; 7 Institute for Quantum Science and Engineering, Texas A&M University, College Station, TX 77843, USA

**Keywords:** quantum steering, bell nonlocality, EPR paradox, decoherence, entangled states, 03.67.-a, 03.65.Yz, 03.65.Ud

## Abstract

By using the Born Markovian master equation, we study the relationship among the Einstein–Podolsky–Rosen (EPR) steering, Bell nonlocality, and quantum entanglement of entangled coherent states (ECSs) under decoherence. We illustrate the dynamical behavior of the three types of correlations for various optical field strength regimes. In general, we find that correlation measurements begin at their maximum and decline over time. We find that quantum steering and nonlocality behave similarly in terms of photon number during dynamics. Furthermore, we discover that ECSs with steerability can violate the Bell inequality, and that not every ECS with Bell nonlocality is steerable. In the current work, without the memory stored in the environment, some of the initial states with maximal values of quantum steering, Bell nonlocality, and entanglement can provide a delayed loss of that value during temporal evolution, which is of interest to the current study.

## 1. Introduction

Entanglement is a crucial physical resource in the development of the necessary tasks for the processing and transmission of quantum information (PTQI) [1,2,3,4,5,6,7]. It is a kind of nonlocal correlation that exists in nonseparable quantum states. Specially, correlations as the result of local measurements performed on entangled systems may display nonlocal correlations [8]. The local hidden variable (HV) model constrains measurement statistics for a wide range of systems [9]. The paradox introduced by Einstein et al. [10], contends that the theory of quantum mechanics demonstrates “spooky action at a distance”. Subsequently, Schrödinger elucidated this phenomena with the possibility of taking local measurements to influence a remote subsystem without accessibility to it, a process known as Einstein–Podolsky–Rosen steering (EPR) steering (quantum steering) [11]. EPR steering is a nonlocal correlation in itself and among the different methods for the detection of quantum steering there exists the simplest way to formulate criteria for the correlations between Alice’s and Bob’s measurement statistics. Steerable states are regarded a subtype of entangled states [12]. EPR steering recently received attention in theoretical and experimental research [13,14,15,16,17,18,19]. Quantum channel discrimination [20], key distribution [21], and quantum teleportation [22] all use steerable states. Furthermore, quantum steering is founded on operational explanations [23]. By examining the entropy uncertainty relation [24,25,26], certain inequalities in EPR steering are proven. Several theoretical and practical breakthroughs were published to better comprehend this type of nonlocality [25,26,27,28], where key requirements for quantum steering from diverse perspectives were established [29,30]. The Bell scenario is addressed to demonstrate the Bell inequalities for EPR steering [31]. Moreover, the relationship between the quantum entanglement and uncertainty relation [32,33,34] is taken into account while developing quantum steering criteria [29,30].

Realistic systems are open, which causes the most essential quantum features to be rapidly destroyed. As a result, it is crucial to look at how correlations behave when a quantum system loses its coherence due to interaction with the environment. Because every natural system interacts with its surrounds, researchers are focusing more on the system–environment interaction and understanding the dynamical behavior of various types of correlations. In the literature, the dynamics of quantum entanglement in open systems received a lot of attention. Moreover, several studies looked at the effect of the environment on the quantum discord’s dynamical behavior. In this context, several comparisons were made between the quantum discord and quantum entanglement dynamics of the system of two qubits within the Markovian and non-Markovian environment [35,36,37,38]. In the Markovian dynamics, it was demonstrated that their behavior is entirely different and that discord is more resistant to the decoherence effect than quantum entanglement. Recently, distinct particular characteristics in the dynamics of several types of classical and quantum correlations in the existence of Markovian noise were shown [36,38].

In this article, we use the Born Markovian master equation to examine the temporal development of EPR steering, Bell nonlocality, and quantum entanglement for ECSs. This physical model has a diverse set of quantum states with correlations that vary according to the strength regimes of the optical fields of ECSs. Analytical formulas and numerical representations are provided to show the dependency of the three kinds of correlations on the odd and even ECSs. Generally, we find that the measures of correlations start from their maximal values and decrease with the time. We find that the quantum steering and nonlocality have similar behavior with respect to the photon number during dynamics. Moreover, we find that the ECSs with the steerability can violate the Bell inequality, and not every ECS is steerable with Bell nonlocality. These correlations can be preserved during the evolution and resist against environment, exhibiting an important feature of quantum steerability and nonlocality in the present model. According to the recent researches [39,40,41,42,43,44], our findings from a phenomenological standpoint may be useful in describing the behavior of various kinds of correlations under the influence of the environment.

The structure of the paper is as follows. Section 2 introduces the concepts of EPR steering, nonlocality, and quantum entanglement. In Section 3, we outline the physical model with ECS decoherence and present the results along with a discussion. The final part contains a summary.

## 2. Steering, Bell Nonlocality, and Quantum Entanglement

In this section, we introduce a brief review on the quantum quantifiers including EPR steering, nonlocality, and quantum entanglement. Recently, there was a growing interest in the issue of detecting and characterizing these classes of correlations.

Let us consider ${\hat{x}}^{a}$ (${\hat{x}}^{b}$) and ${\hat{k}}^{a}$ (${\hat{k}}^{b}$) to be observables (continuous) of system *a* (*b*) with outcomes $\left\{x^{a}\right\}$ ($\left\{x^{b}\right\}$) and $\left\{k^{a}\right\}$ ($\left\{k^{b}\right\}$), respectively. Following the references [26,30], by using the positivity of the continuous entropies, Walborn et al. [25] discussed the observables in states that verify local hidden state model (LHSM) $h\left(x^{b}|x^{a}\right)\geq \int d\lambda \rho \left(\lambda \right)h_{q}\left(x^{b}|\lambda \right)$, where λ represents the HV, ρ(λ) is the probability density, and $h_{q}\left(x^{b}|\lambda \right)$ is the continuous Shannon entropy that are originated by the probability density. The parameter *q* indicates that the probability density obtained from a system directed by the HV λ. Walborn et al. demonstrated that any states having a LHSM in momentum and position should verify

(1) $$h\left(k^{b}|k^{a}\right)+h\left(x^{b}|x^{a}\right)\geq \log\left(\pi e\right).$$

Following that, it was discovered that identical reasoning considered to build LHSM constraints for continuous observables may also be employed to develop LHSM constraints for observables that are discrete [30]. The related Local hidden states constraint for discrete variables may be obtained by utilizing the positivity of the relative entropy: $H\left(R^{b}|R^{a}\right)\geq \sum_{\lambda }P\left(\lambda \right)H_{q}\left(R^{b}|\lambda \right)$, where ${\hat{R}}_{i}^{a}$ (${\hat{S}}_{i}^{a}$) and ${\hat{R}}_{i}^{b}$ (${\hat{S}}_{i}^{b}$) are discrete observables with outcomes $\left\{R_{i}^{a}\right\}$ ($\left\{S_{i}^{a}\right\}$) and $\left\{R_{i}^{b}\right\}$ ($\left\{S_{i}^{b}\right\}$), respectively, and the index *i* runs from 1 to the total number of distinct eigenstates. $H_{q}\left(R^{b}|\lambda \right)$ defines the Shannon entropy (discrete) of $P_{q}\left(R^{b}|\lambda \right)$ with $H\left(R\right)=-\sum_{i}P\left(R_{i}\right)\ln P\left(R_{i}\right)$. Therefore, entropic steering inequality in the case of discrete variables is introduced by [30]:

(2) $$H\left(R^{b}|R^{a}\right)+H\left(S^{b}|S^{a}\right)\geq \log\left(\Omega^{b}\right).$$

Here, $\Omega^{b}$ represents the value $\Omega \equiv \min_{i,j}\left(1/|\langle R_{i}|S_{j}\rangle {|}^{2}\right)$ with $\left\{|R_{i}\rangle \right\}$ and $\left\{|S_{i}\rangle \right\}$ design, respectively, the observable eigenbases in the *N*-dimensional Hilbert space. The steering inequality expresses [30] as

(3) $$H\left(\sigma_{x}^{b}|\sigma_{x}^{a}\right)+H\left(\sigma_{y}^{b}|\sigma_{y}^{a}\right)+H\left(\sigma_{z}^{b}|\sigma_{z}^{a}\right)\geq 2.$$

When the inequality is broken, quantum steering is proven. We consider the *X*-state of the system of two qubits

(4) $$W_{X}=\left(\begin{array}{cccc} W_{11} & 0 & 0 & W_{14}\\ 0 & W_{22} & W_{23} & 0\\ 0 & W_{23} & W_{33} & 0\\ W_{14} & 0 & 0 & W_{44} \end{array}\right),$$

where $W_{ij}$ are considered as real numbers. The provided state $W_{X}$ in Equation (4) may be represented in Bloch decomposition as

(5) $$W_{X}=\frac{1}{4}\left(1^{a}⊗1^{b}+\vec{r}\cdot {\vec{\sigma }}^{a}⊗1^{b}+1^{a}⊗\vec{s}\cdot {\vec{\sigma }}^{b}+\sum_{j=1}^{3}c_{j}\sigma_{j}^{a}⊗\sigma_{j}^{b}\right),$$

where $\vec{s}$ and $\vec{r}$ represent Bloch vectors and $\sigma_{j}^{a,b}$ are the Pauli matrices. Thereafter, utilizing the state $W_{X}$ in Equation (4) and the formulation of steering inequality in Equation (3), the expression of steering inequality is provided by [41]

(6) $$\begin{array}{ccc} \; & \; & \sum_{j=1,2}\left[\left(1+c_{j}\right)\log_{2}\left(1+c_{j}\right)+\left(1-c_{j}\right)\log_{2}\left(1-c_{j}\right)\right]-\left(1+r\right)\log_{2}\left(1+r\right)-\left(1-r\right)\log_{2}\left(1-r\right)\\ & + & \frac{1}{2}\left(1+c_{3}+r+s\right)\log_{2}\left(1+c_{3}+r+s\right)+\frac{1}{2}\left(1+c_{3}-r-s\right)\log_{2}\left(1+c_{3}-r-s\right)\\ & + & \frac{1}{2}\left(1-c_{3}-r+s\right)\log_{2}\left(1-c_{3}-r+s\right)+\frac{1}{2}\left(1-c_{3}+r-s\right)\log_{2}\left(1-c_{3}+r-s\right)\leq 2, \end{array}$$

where

(7) $$\begin{array}{ccc} c_{1} & = & 2(W_{23}+W_{14})\\ c_{2} & = & 2(W_{23}-W_{14})\\ c_{3} & = & W_{11}+W_{44}-W_{22}-W_{33}\\ r & = & W_{11}+W_{22}-W_{33}-W_{44}\\ s & = & W_{11}-W_{22}+W_{33}-W_{44} \end{array}$$

The diagonal and antidiagonal components of the $W_{X}$ state for *i* equals *j* and *i* is different from *j*, respectively, are represented by $W_{ij}$. Cavalcanti, Jones, Wiseman, and Reid (CJWR) devised an inequality to determine if a quantum state is steerable [29]. Recently, Cavalcanti et al. presented additional parameters that enable for measurement of EPR steering [45]. Subsequently, R. M. Angelo et al. proposed a measure of quantum steering based on the maximum violation of the CJWR steering inequality [46].

To investigate the Bell nonlocality, we introduce the Bell Clauser–Horen–Shimony–Holt model (CHSH). According to the Horodecki criteria [9], $B=2\sqrt{\max_{i<j}\left(\mu_{i}+\mu_{j}\right)}$ with i,j=1,2,3. Here, $\mu_{i}$ represent the three eigenvalues of $U=T^{t}T$ with T is a real matrix that can be evaluated from the $t_{ij}$ coefficients [9,47]

(8) $$t_{ij}=\mathrm{Tr}\left(W\sigma_{i}⊗\sigma_{j}\right)$$

where the matrix $T^{t}$ is the transposition of the matrix T. They are introduced by

(9) $$\begin{array}{ccc} \mu_{1} & = & 4{\left(|W_{14}\left|+\right|W_{23}|\right)}^{2}\;\mu_{2}=4{\left(|W_{14}\left|-\right|W_{23}|\right)}^{2};\\ & \; & \mu_{3}={\left(W_{11}-W_{22}-W_{33}+W_{44}\right)}^{2}. \end{array}$$

$\mu_{1}$ is greater than $\mu_{2}$, and so the largest violation of the Bell–CHSH inequality is provided by [41]

(10) $$B=2\max\left\{B_{1},B_{2}\right\},\;B_{1}=\sqrt{\mu_{1}+\mu_{2}},\;B_{2}=\sqrt{\mu_{1}+\mu_{3}}.$$

To demonstrate entanglement in the TMCSS, we examine the entanglement of formation (EOF) introduced by [48,49]

(11) $$E\left(W\right)=H\left[\frac{1+\sqrt{1-C^{2}\left(W\right)}}{2}\right].$$

Here, the function H is given by

(12) $$H\left(\alpha \right)=-\alpha \log_{2}\alpha -\left(1-\alpha \right)\log_{2}\left(1-\alpha \right),$$

and the concurrence is expressed as

(13) $$C\left(W\right)=\max\left\{0,\sqrt{\beta_{1}}-\sqrt{\beta_{2}}-\sqrt{\beta_{3}}-\sqrt{\beta_{4}}\right\},$$

where $\beta_{i}$ are the eigenvalues of $W\tilde{W}$ that are listed in decreasing order. $\tilde{W}$ designs the time-reversed density operator

(14) $$\tilde{W}=\left(\sigma_{y}⊗\sigma_{y}\right)W^{*}\left(\sigma_{y}⊗\sigma_{y}\right)$$

where $W^{*}$ denotes the complex conjugate of the operator *W*, whereas $\sigma_{y}$ indicates the Pauli matrix of *y*. Entanglement is measured in terms of E, which ranges from 0 for factorizable states to 1 for maximally entangled states.

## 3. Physical System and Dynamics

The quantum system that we are proposing to study has significant applications in several tasks of PTQI. Its richer structure shows important advantages in different tasks of PTQI [50], where many tasks of PTQI require ECSs. Theoretically, these states can be generated by using a cat state, $|cat\rangle ={\left[2\left(1\pm e^{-{4|\xi |}^{2}}\right)\right]}^{-\frac{1}{2}}\left(|\sqrt{2}\xi \rangle \pm |-\sqrt{2}\xi \rangle \right)$, and a beam splitter device, where $|\pm \xi \rangle =e^{-\frac{{\left|\xi \right|}^{2}}{2}}\sum_{n=0}^{\infty }{\left(\pm \xi \right)}^{n}/\sqrt{n!}|n\rangle$ defines the Glauber coherent state with ξ∈C and |n〉 represents the Fock state. These quantum states define the simplest states of continuous variables that are the closest analogues to the classical light field and present the Poisson distribution of photons, and have potential applications in several domains such as condensed matter physics, quantum optics, and statistical mechanics [6,51]. They have well-defined phase and amplitude, and their uncertainties are limited to the Heisenberg uncertainty principle.

The ECSs introduced in this manuscript are in the form of [52,53,54,55,56,57,58,59,60,61,62]

(15) $$|\psi^{\pm }\rangle =N^{\pm }\left({|\xi \rangle }_{1}{|-\xi \rangle }_{2}{\pm |-\xi \rangle }_{1}{|\xi \rangle }_{2}\right)$$

where $N^{\pm }=1/\sqrt{2\pm 2\exp(-4\xi^{2})}$ are the normalization constant and |±ξ〉 designs a coherent state containing $\xi^{2}$ photons on the average. For simplicity, the complex amplitude ξ is considered as a real parameter and we note that $|\psi^{+}\rangle$ ($|\psi^{-}\rangle$) is an even (odd) ECSs with even (odd) numbers of photons.

When the vacuum environment affects the ECSs, the quantum states decohere and merge to form mixed states $\rho^{}\left(t\right)$. Under the Born–Markov approximation, evolution of the density matrix occurs over time by

(16) $$\frac{\partial W^{\pm }\left(t\right)}{\partial t}=\hat{L}W^{\pm }\left(t\right)+\hat{J}W^{\pm }\left(t\right),$$

where $\hat{L}W^{\pm }\left(t\right)=-\sum_{i=1}^{2}\frac{\gamma }{2}\left({\hat{a}}_{i}^{†}{\hat{a}}_{i}W^{\pm }\left(t\right)+W^{\pm }\left(t\right){\hat{a}}_{i}^{†}{\hat{a}}_{i}\right)$, $\hat{J}W^{\pm }\left(t\right)=\sum_{i=1}^{2}\gamma {\hat{a}}_{i}W^{\pm }\left(t\right){\hat{a}}_{i}^{†}$, γ represents the decay rate, and ${\hat{a}}_{i}$ (${\hat{a}}_{i}^{†}$) defines the annihilation (creation) operator of the single mode *i*. The formal solution of the master equation can be expressed as $W^{\pm }\left(t\right)=\exp\left[\left(\hat{L}+\hat{J}\right)t\right]W^{\pm }\left(0\right)$, where $W^{\pm }\left(0\right)$ is density matrix at t=0. Although there are several ways to study systems, the approach of the master equation is widely utilized and is very suitable in the fields of quantum optics. Assuming a zero-temperature reservoir, the dynamics of $W^{\pm }$ described by

(17) $$\begin{array}{cc} W^{\pm }\left(t\right)= & {\left(N^{\pm }\right)}^{2}{\{|\xi \left(t\right)\rangle }_{1}{|-\xi \left(t\right)\rangle }_{2}\langle \xi \left(t\right)|\langle -\xi \left(t\right)|\\ & {+|-\xi \left(t\right)\rangle }_{1}{|\xi \left(t\right)\rangle }_{2}\langle -\xi \left(t\right)|\langle \xi \left(t\right)|\\ & \pm e^{-4\xi^{2}r^{2}}\left({|\xi \left(t\right)\rangle }_{1}{|-\xi \left(t\right)\rangle }_{2}\langle -\xi \left(t\right)|\langle \xi \left(t\right)|+h.c.\right)\}, \end{array}$$

where ${|\pm \xi \left(t\right)\rangle }_{i}={|\pm \xi e^{-\frac{\gamma t}{2}}\rangle }_{i}$ (i=1,2) and the superscript +(−) is associated to the even (odd) ECSs. The normalized time *r* is related to the time *t* by $r=\sqrt{1-e^{-\gamma t}}$. The density operator $W^{\pm }\left(t\right)$ can be given in the basis set ${|\pm \rangle }_{i}=n^{\pm }\left({|\xi \left(t\right)\rangle }_{i}\pm {|-\xi \left(t\right)\rangle }_{i}\right)$ as

(18) $$W^{\pm }\left(r\right)=\left(\begin{array}{cccc} W_{11}^{\pm }\left(r\right) & 0 & 0 & W_{14}^{\pm }\left(r\right)\\ 0 & W_{22}^{\pm }\left(r\right) & W_{23}^{\pm }\left(r\right) & 0\\ 0 & W_{23}^{\pm }\left(r\right) & W_{33}^{\pm }\left(r\right) & 0\\ W_{14}^{\pm }\left(r\right) & 0 & 0 & W_{44}^{\pm }\left(r\right) \end{array}\right)$$

where

(19) $$\begin{array}{ccc} W_{11}^{\pm }\left(r\right) & = & \frac{{\left(N^{\pm }\right)}^{2}}{8}\left[\frac{1\pm e^{-4\xi^{2}r^{2}}}{{\left(n^{+}\right)}^{4}}\right]\\ W_{22}^{\pm }\left(r\right) & = & W_{33}^{\pm }\left(r\right)=-W_{23}^{\pm }\left(r\right)=\frac{{\left(N^{\pm }\right)}^{2}}{8}\left[\frac{1\mp e^{-4\xi^{2}r^{2}}}{{\left(n^{+}n^{-}\right)}^{2}}\right]\\ W_{44}^{\pm }\left(r\right) & = & \frac{{\left(N^{\pm }\right)}^{2}}{8}\left[\frac{1\pm e^{-4\xi^{2}r^{2}}}{{\left(n^{-}\right)}^{4}}\right]\\ W_{14}^{\pm }\left(r\right) & = & -\frac{{\left(N^{\pm }\right)}^{2}}{8}\left[\frac{1\pm e^{-4\xi^{2}r^{2}}}{{\left(n^{+}n^{-}\right)}^{2}}\right]. \end{array}$$

**Figure 1:**
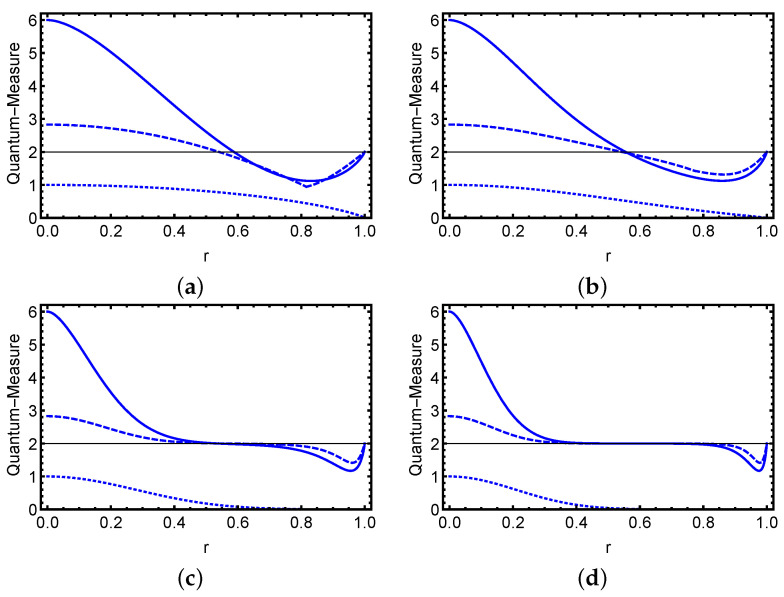
For various values of the parameter ξ, the three classes of correlations of the initial odd state are plotted in terms of the normalized time *r*. Panel (**a**) is for ξ=0.05, panel (**b**) is for ξ=0.8, panel (**c**) is for ξ=1.5, and panel (**d**) is for ξ=2. The time evolution of the steering inequality is shown by the solid blue line, the time development of the Bell–CHSH inequality is represented by the dashed blue line, and the time evolution of the formation entanglement is represented by the dotted blue line.

**Figure 2:**
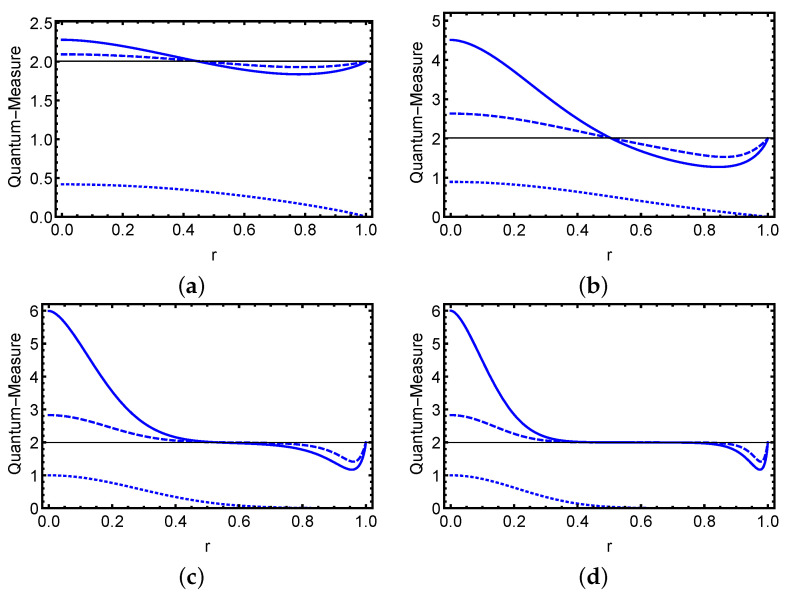
For various values of the parameter ξ, the three classes of correlations of the initial even state are plotted in terms of the normalized time *r*. Panel (**a**) is for ξ=0.4, panel (**b**) is for ξ=0.8, panel (**c**) is for ξ=1.5, and panel (**d**) is for ξ=2. The time evolution of the steering inequality is shown by the solid blue line, the time development of the Bell–CHSH inequality is represented by the dashed blue line, and the time evolution of the formation entanglement is represented by the dotted blue line.

Using the Equations (6), (10) and (11), we are able to show the influence of decoherence on the performance and relationship among quantum steering, Bell nonlocality and quantum entanglement for different strength regimes of the input optical fields. In Figure 1 and Figure 2, we show the temporal variation of the three types of correlations as a function of the normalized time *r* for various photon number $\xi^{2}$ values in the case of even (odd) ECSs that are entangled (maximally entangled) states in the 2⊗2 Hilbert space at initial instant r=0. The solid line represents the variation of the steering inequality, the dashed line represents the Bell inequality, and the dotted line represents formation entanglement. In general, we find that as time passes, the EPR steering, Bell nonlocality, and quantum entanglement diminish progressively. Furthermore, the effect of decoherence demonstrates that quantum steering and nonlocality behave similarly in terms of photon number values. The three variables are first reduced from their maximum values at r=0, where the ECS is maximally steerable with optimally steering, Bell nonlocality with Tsirelson’s limits, and we obtain a maximal entangled state. If their quantum state is maximally entangled during the dynamics, the steering and Bell nonlocality are greater. Following that, the three correlations diminish with time, and the initial even and odd ECSs reach a mixed state with zero entanglement and no violation of the EPR steering or Bell inequalities. We can observe that the quantum steering and nonlocality disappear at approximately the same time, where the entanglement disappears after that time. This shows that all steerable states can violate the Bell inequality, and that for some ranges of the decoherence, the two mode state is entangled and cannot violate the seteering and Bell inequalities. The advantage of the normalized time is that the decoherence process can be studied completely, between (0, *∞*) using r in (0, 1). As a result, an examination of these quantum system measures in the presence of decoherence in the limit of very large *t* is highly sought. This issue is also considered in the current effort.

To investigate what occurs in the situation of mixed states in relation to different optical field intensity regimes, we demonstrate the dynamical behavior of the three types of correlations for different photon number values in odd and even states. We can observe that a suitable choice of photons number can result in time-delay control of quantifiers loss. We can observe that the steerability, Bell nonlocality, and entanglement of the initial state can be destroyed by increasing the photons number and we have small values of time for which the two-mode state cannot steer with the absence of Bell nonlocality and entanglement. Furthermore, we can show that for small values of parameter ξ, the steerability of odd states is stronger than the steerability of even states, and the steerability (or Bell nonlicality) of these states coincides when the photons number gets substantially big. In this situation, without the memory encoded in the environment, certain of the initial states with maximal values of quantum steering, Bell nonlocality, and entanglement can provide a delayed loss of that value during the temporal evolution, which is of interest to the current study.

## 4. Conclusions

By using the Born Markovian master equation, we studied the relevance among the Einstein–Podolsky–Rosen (EPR) steering, Bell nonlocality, and quantum entanglement of ECSs under decoherence. We displayed the dynamical behavior of the three classes of correlations for different regimes of the input optical fields. We compared the dynamics of the correlations for the case of odd and even ECSs. We observed that correlation measures begin at their maximum and decline with time. We showed that quantum steering and nonlocality dynamics behave similarly in terms of photon number. We also illustrated that ECSs with steerability can defy the Bell inequality, and that not all ECSs with Bell nonlocality are steerable. We demonstrated that a suitable choice of photons number can dictate time-delay control of quantifiers loss. We showed that reducing the number of photons improves the initial state’s correlations, and we demonstrated that the two-mode state can not steer in the absence of Bell nonlocality and entanglement for a short period of time. In the current work, without the memory recorded in the environment, some initially states with maximal values of quantum steering, Bell nonlocality, and entanglement can provide a delayed loss of that value during temporal evolution, which is of interest to the current study. 

## Data Availability

Not applicable.
